# Stroke in Pregnancy: A Case Report

**DOI:** 10.31729/jnma.8112

**Published:** 2023-04-30

**Authors:** Ravi Kumar Shah, Arushi Jaiswal, Rehana Mushtaq, Sana Ansari, Jagat Prasad Deep

**Affiliations:** 1 Department of Obstetrics and Gynaecology, National Medical College and Teaching Hospital, Birgunj, Parsa, Nepal; 2 Department of Anaesthesia, National Medical College and Teaching Hospital, Birgunj, Parsa, Nepal

**Keywords:** *case reports*, *hypertension*, *intracerebral haemorrhage*, *pregnancy*, *stroke*

## Abstract

Reported here is a rare near-miss case of stroke in pregnancy presented to the Department of Obstetrics and Gynaecology. A 38 years gravida 8 was referred from a private hospital on 18 November 2022 with hemorrhagic stroke, a known case of chronic hypertension at 37 weeks of gestation with previous cesarean section and acute kidney injury. At a private hospital computed tomography head was done it showed intracerebral haemorrhage. On cesarean, intraoperatively it was a live female with thick meconium. The patient was kept in intensive care with a mechanical ventilator, antihypertensives, antibiotics, and analgesics. Serum creatinine was increasing daily. Suture was cut on the 7^th^ postoperative and two times dialysis was done on the 8^th^ and 9^th^ postoperative days. Stroke in pregnancy is a rare diagnosis and it could have been prevented by regular antenatal visits and early referral antenatally along with a multidisciplinary approach.

## INTRODUCTION

A stroke is a neurological emergency that is caused by acute focal injury of the central nervous system resulting from a vascular cause such as cerebral infarction, cerebral vein thrombosis, intracranial haemorrhage, or subarachnoid. Strokes caused by pregnancy occur at a rate of 34 per 100,000 births and are the second leading cause of death.^1^ Pregnancy-related strokes are also the greatest cause of serious long-term disability following pregnancy. Pregnancy hypertensive illnesses such as preeclampsia and eclampsia, as well as prothrombotic condition that increases the incidence of arterial and venous thrombosis, are risk factors for stroke.^2^

## CASE REPORT

**Figure 1:**
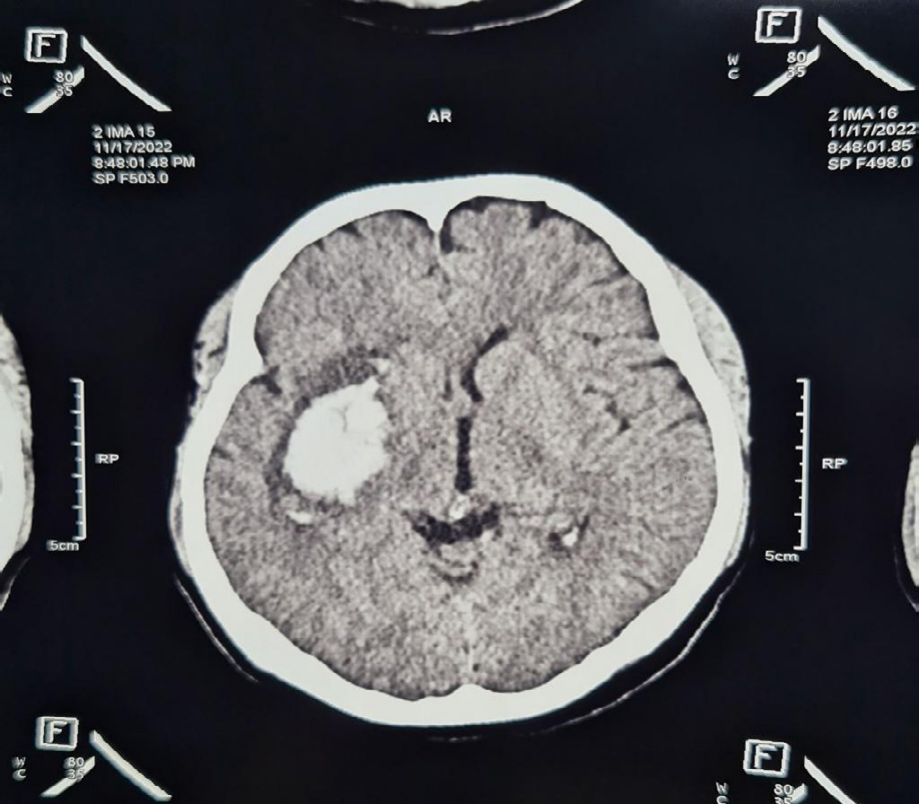
Noncontrast computed tomography head - Intracranial Hemorrhage.

A 38 years old grand multipara, known case of hypertension was referred from a private hospital to our tertiary centre on 18 November 2022 with a hemorrhagic stroke. According to history, she was a known case of hypertension for 3 years and was not taking any antihypertensive and it was an unplanned pregnancy spontaneous, and unbooked pregnancy. Her pregnancy was going uneventful till yet then she developed a headache which was followed by a sudden loss of consciousness after 2 days. For these complaints, she was initially taken to a private hospital where her non-contrast computed tomography of the head was carried out showing a right-sided intracranial haemorrhage (Figure 1).

**Figure 2:**
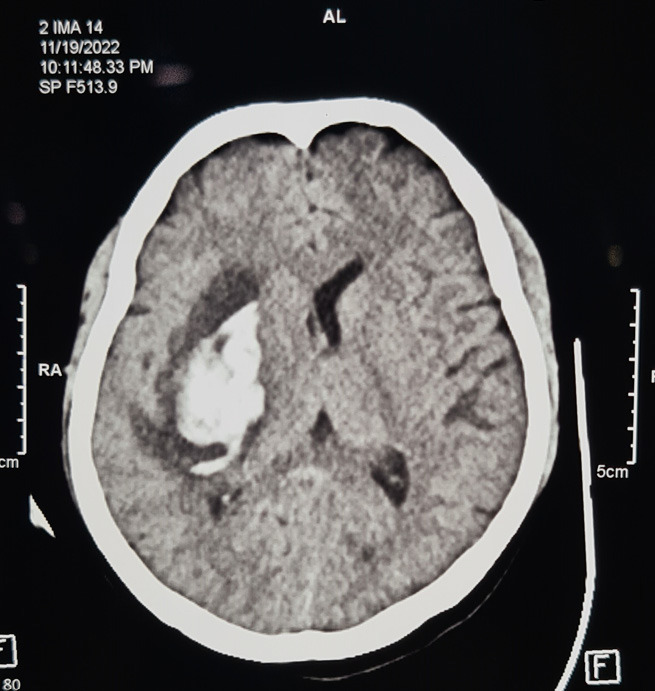
Noncontrast computed tomography head showing intracerebral haemorrhage with mass effect.

After giving first aid treatment she was referred to our center for better management. Here, on examination, the patient was confused and drowsy. Glasgow coma scale of 11 (E2 V4 M5), blood pressure of 160/110 mmHg. On CNS examination muscle power was decreased on the left side of the body (2/5). However, it was normal on the right side (5/5). On per abdomen examination, Pfannenstiel scar was there, symphysiofundal height was 34 cm with a longitudinal lie, and 2 mild contractions in 10 minutes. Fetal heart sound was 140 beats per minute. On per vaginal examination, the cervix was minimally effaced and thick meconium-stained liquor was present. Blood investigations were within normal range except for serum creatinine level which was high. Here patient was approached multidisciplinary along with medicine, anaesthesia and neurosurgery, paediatric and physiotherapy department. Her emergency lower segment caesarean section with tubal ligation was performed under general anaesthesia. Intraoperatively minimal adhesion was present, the bladder was normal, the lower uterine segment was well formed, the previous scar was intact, liquor scanty and thick meconium stained, the baby was in left occipitoanterior position, placenta fundoanterior, bilateral tubes and ovaries were normal and the tubal ligation was done with estimated blood loss of 150 ml. Her fetal outcome was an alive baby girl weighing 1.5 kg with an APGAR score of 7 and 8 in 1 and 5 minutes respectively. Her intraoperative period remained uneventful with a urine output of 200 ml. Following caesarean section, the patient was shifted to the intensive care unit and kept on mechanical ventilation. Here in the ICU patient was further managed conservatively. Along with routine postoperative care, antihypertensive drugs include labetalol, glyceryl trinitrate, prazosin and amlodipine. Her non-contrast computed tomography head was repeated after 2 days of having the first scan which showed well defined intra-axial hyperdense area measuring approximately 4.1×2.6×4.8 (volume ~ 21.4cc) with mild perilesional oedema noted in the right centrum semi-ovale, corona radiata, lentiform nucleus, internal capsule and temporal lobe causing mass effect as evident by ipsilateral ventricle and midline shift towards contralateral side by 2.1 mm likely intracerebral haemorrhage with mass effect (Figure 2).

On the 4^th^ postoperative day, the patient was weaned off from mechanical ventilation and was put on T-piece maintaining oxygen saturation at 5 litres of oxygen. She was further kept on T-piece till the 7^th^ postoperative day when she was extubated and sutures were also removed on the same day.

In her postoperative period, her blood investigation remained normal except for blood urea and serum creatinine which continued to rise. For that, she underwent Haemodialysis twice that is on days 8^th^ and 9^th^ postoperatively. On the 10^th^ postoperative day, the patient was shifted from ICU to the general ward of neurosurgery where she was rehabilitated for the rest of the days and then discharged on the 26^th^ postoperative day which is with the advice to be in regular follow-up.

## DISCUSSION

The American Heart Association and the American Stroke Association (AHA/ASA) broadly define "stroke" to include ischaemic stroke caused by an arterial or venous infarction of the central nervous system as well as haemorrhagic stroke, which includes nontraumatic intracerebral haemorrhage (ICH) and subarachnoid haemorrhage (SAH).^3^ Globally, 37% of all cardiovascular fatalities in women are caused by strokes.^4^ Pregnancy-related hypertensive disorders are responsible for 20-50% of all peripartum strokes.^5,6^ Preexisting, gestational, or secondary to preeclampsia or eclampsia, pregnancy-related hypertension can occur. Increased capillary permeability, platelet activation, and thrombin/fibrin production, as well as diffuse arterial vasospasm and severe hypertension, are all potential risk factors for stroke in this hypertensive disorder. Recent estimates put the prevalence of maternal strokes at approximately 30 per 100,000 pregnancies.^7^ Although stroke can happen at any time during pregnancy, the highest risk times are the third trimester and the first few weeks after delivery (within the first 6 weeks postpartum).^8^ Strokes caused by pregnancy are frequently linked to mothers over the age of 35.^2^ Diabetes, hypertension, dyslipidemia, and smoking continue to be significant traditional medical stroke risk factors during pregnancy. The ability to distinguish between ischemic and haemorrhagic episodes and confirm or rule out a diagnosis of stroke depends on quick neuroimaging and rapid presentation. The fetus is exposed to extremely low amounts of radiation during all routine neuroimaging procedures.^9^ For the diagnosis of stroke, MRI and CT imaging are both options. To provide the best outcome and reduce the risk of haemorrhage, thrombolytic treatment must be administered promptly because it is time-dependent. Placental haemorrhage or abruption may occur despite the fact that rt-PA is a large molecule that does not cross the placental circulation.^10^ Thrombolysis, on the other hand, should only be used for life-threatening situations because it can cause uncontrolled bleeding in the postpartum period, especially after a caesarean section. After an ischemic stroke, a thrombectomy has a significant impact on survival and functional recovery. Pregnancy should not impede treatment after a haemorrhagic stroke diagnosis, and endovascular procedures can be performed if necessary. Depending on the best option for the patient, ruptured aneurysms can be coiled or clipped. Depending on the modality, ruptured arteriovenous malformations can be resected or embolized.^2^ Therefore, a reasonable goal for the acute setting is a blood pressure of 160/110 mm Hg. Osmotic diuresis, permissive hypocapnia, and a decompressive craniotomy may be necessary if intracranial hypertension persists.^11^ In the setting of acute care, stroke rehabilitation should begin early. Long-term functional outcomes have been linked to therapy of sufficient intensity, task-oriented training, and excellent team coordination.

Women who suffer a stroke during pregnancy are more likely to suffer from long-term complications even after giving birth, and they continue to require the assistance of a multidisciplinary team of healthcare providers, including social workers and mental health professionals. The burden of stroke cannot be significantly reduced by the treatment provided by even the most advanced and best healthcare system. As a result, the word "prevention is better than cure" holds true for stroke. In conclusion, although strokes during pregnancy are uncommon, their incidence is rising,^12^ possibly as a result of rising hypertension in young women prior to and during childbearing. Therefore, it is essential to identify risk factors for stroke during pregnancy in order to prevent this uncommon but frequently fatal condition. Despite its rarity, stroke is a significant cause of pregnancy-related mortality and morbidity.^13^ A multidisciplinary stroke unit with specialized medical care, optimized treatment protocols, coordinated rehabilitation services, and patient education should be used to manage all patients. However, in this instance, the complication was discovered during the late stage due to inadequate awareness, resources, and antenatal checkups also patient was not followed up after discharge till now which resulted in the rarest complication of stroke in pregnancy.
